# Systemic Cytokine and Chemokine Profiles in Individuals With *Schistosoma mansoni* Infection and Low Parasite Burden

**DOI:** 10.3389/fimmu.2018.02975

**Published:** 2018-12-18

**Authors:** Vanessa N. Castro, Jailza L. Rodrigues, Diogo T. Cardoso, Samira D. Resende, Fernanda C. Magalhães, Dayane C. Souza, Maira H. Requeijo, Deborah Negrão-Corrêa, Stefan M. Geiger

**Affiliations:** ^1^Department of Parasitology, Federal University of Minas Gerais (UFMG), Belo Horizonte, Brazil; ^2^Faculdade da Saúde e Ecologia Humana (FASEH) Vespasiano, Belo Horizonte, Brazil

**Keywords:** *Schistosoma mansoni*, humans, low parasite load, immunological markers, CCL17

## Abstract

Intestinal schistosomiasis, caused by the parasitic trematode *Schistosoma mansoni*, is a chronic disease and the prolonged and continuous exposure to *S. mansoni* antigens results in a deviation of the host's immune response. For diagnosis, the Kato-Katz (KK) method is recommended, however, this method showed low accuracy in areas of low endemicity. This study aimed to characterize the cytokine and chemokine profile of individuals with an extremely low parasite load (<4 eggs per gram of feces), e.g., individuals who were detected by alternative parasitological methods, such as the saline gradient and/or Helmintex®. In order to search for immunological markers for infection, the immunological profile in serum samples of these individuals was then compared with patients detected with the KK method and with a higher parasite load and with individuals repetitively negative by extensive stool exams. The study was conducted in Northern Minas Gerais in a rural area of the Municipality of Januária. Serum samples of a total of 139 parasitologically well-characterized individuals were assessed for the following immunological markers by commercially available immunoassays: TNF-α, IL-1β, IL-6, IL-17A, IL-5, IL-10, IL-13, IL-33, IL-27, CCL3, CCL5, CXCL10, CCL11, and CCL17. As a result, there were no significant differences in concentrations or frequencies for immunological markers between egg-negative individuals or individuals with ultra-low (<4 epg) or low (4–99 epg) parasite loads. However, we found significant correlations between egg counts and eosinophil counts and between egg counts and IL-1β or TNF-α concentrations. The most striking alterations were found in individuals with the highest parasite load (≥100 epg). They had significantly higher TNF-α concentrations in serum when compared with individuals with a low parasite load (4–99 epg) and CCL17 concentrations were significantly elevated when compared with egg-negative individuals. Radar diagrams of frequencies for cytokine and chemokine responders in each infection group confirmed a distinct profile only in the infection group with highest parasite loads (≥100 epg).

## Introduction

Schistosomiasis is a-chronic disease with estimates of more than 250 million infected people. Of these, the biggest part, about 201.5 million infected individuals, lives in Africa (1–3). In Brazil, the only species found is *Schistosoma mansoni*. Here, as in other parts of the world, the disease is still considered a public health problem and, most recently, it was estimated that around 1.5 million people are infected (4) and 25 million people live in endemic areas at risk of infection (5, 6). One of the main advances in the control of schistosomiasis was the implementation of the Brazilian Schistosomiasis Control Program (BSCP) in the 1970s, following the recommendations of the World Health Organization. One of the pillars of BSCP is the readily detection of infected individuals by large scale stool examinations, using the Kato-Katz (KK) method (7) and the subsequent treatment, in order to minimize mortality and morbidity of the affected population (8). Nowadays, with the advances of schistosomiasis control worldwide, the goals of WHO have moved from reduction of mortality rates and reduce morbidity to transmission control and even eradication in some areas (9). Other positive effects of the ongoing efforts on schistosomiasis control include significant reduction of prevalences and of individual parasite loads in endemic populations (5). As a consequence, most of the infected individuals in Brazil harbor low parasite loads, which are hardly detected by the commonly applied parasitological methods (10–12), as initially recommended by the WHO (9). However, if health services seek to advance to transmission control or even eradication, more sensitive diagnostic methods have to be integrated in endemic areas, as it was shown by Oliveira et al. (13).

During their development in the human body, the different parasitic stages of schistosomes induce significant alterations in the immune response, both during the acute and chronic phase of infection (14–17). In the course of the infection, the parasite-specific immune response is modulated from a type 1, inflammatory and cell-mediated, to a type 2, and more antibody-dependent immune response (15, 17–19). In schistosomiasis endemic areas, most of the infected individuals exhibit the intestinal asymptomatic form of the disease and are normally detected already during the chronic phase of infection (18). The majority of these studies on immune responses in humans are based on *in vitro* stimulation of peripheral blood mononuclear cells (PBMC) with schistosome antigens and the detection of intracellular transcription of cytokine or chemokine genes or detection of secretion patterns in cell culture supernatants. Even in stimulated PBMC cultures, such cytokine and chemokine secretion patterns sometimes are subtle and depend on the parasite load and clinical status of the individuals (20, 21) or may even be influenced by co-infections (22). Nevertheless, studies with different schistosome species (23–25) and studies on co-infections with soil-transmitted helminths and *S. mansoni* (26) indicated that systemic inflammatory or chemokine markers and/or type 2 responses might be used as surrogate markers of infection and might be of value for diagnostic purposes.

Therefore, in the present study, we evaluated the serum cytokine and chemokine profile of *S. mansoni*-infected individuals from an endemic area in Brazil in the search for surrogate immunological markers for infection. Here, a special attention was drawn to individuals with medium to low parasite burdens, where common parasitological procedures may be negative and present with reduced sensitivities.

## Materials and Methods

### Ethical Standards

This study was carried out in accordance with the Resolution CNS N° 466/12 from the National Brazilian Research Council and was approved by the Ethics Committee of Centro de Pesquisas René Rachou (Fiocruz) and by the Ethics Committee of the Federal University of Minas Gerais,and is registered at the National Brazilian Platform for Research with Human Subjects under the following number: CAAE # 21824513.9.0000.5091 with written informed consent from all subjects. In the case of minors, additional written informed consent was obtained from their parents or guardians. All subjects gave written informed consent in accordance with the Declaration of Helsinki.

### Study Population

The study was conducted in the district of Brejo do Amparo, Municipality of Januária, about 600 km north of the capital Belo Horizonte, State of Minas Gerais. According to the local health authorities, the local population at the rural communities Corregos Santana, Tocantins, and Pé da Serra did not participate in any schistosomiasis control campaign during the last 2 years prior to the beginning of the study.

In this rural population, the initial prevalence for *S. mansoni* infection after parasitological screening with the KK method, as recommended by the World Health Organization (9), was 20.4%. However, after extensive parasitological examinations with different methods, such as up to 18 KK slides from three fecal samples, saline gradient, and Helmintex®, the prevalence reached 45.9% (13).

A longitudinal study was initiated in the area, where ~250 individuals were examined by parasitological methods (13). Individuals with a positive result for *S. mansoni* eggs were examined again 30 days after treatment with praziquantel, where parasitological cure was confirmed. Further follow-up visits took place for the whole population at several time points after treatment.

Out of a total population of ~270 individuals, 250 subjects signed the consent forms, but only 139 patients provided the required three fecal samples for thorough parasitological examinations. For the present immunological study, 139 individuals were enrolled, of which 113 were egg-positive for *S. mansoni* infection. Twenty-six egg-negative individuals served as uninfected controls and were negative for any intestinal protozoan or helminth parasite in extensive parasitological stool examinations, as stated above.

Blood samples (5 ml EDTA tubes) were drawn from these 139 individuals and complete hemograms were performed for each participant by a commercial clinical laboratory (Millenium, Januária). For serum collection, 5–10 ml of venous blood was collected in siliconized tubes. After clotting samples were centrifuged at 2.000 rpm for 15 min and obtained serum samples were aliquoted and stored at −20°C at the field laboratory in Januária. Subsequently, these serum samples were transferred to the main university laboratory and stored at −80°C, until used in immunological assays. From the 113 *S. mansoni*-infected and treated individuals, 78 cured individuals provided blood and were included in the follow-up immunological study at 3 months post-treatment. The extensive parasitological exams, as described in more detail below, allowed us to classify the individuals from the endemic area into different groups and investigate and compare their immunological profiles in the peripheral blood. The classification of the parasite loads based on KK results in the different infection groups was done according to the WHO (9). However, mean individual epg values were calculated from a total of six KK slides from three fecal samples, as recommended by others (9, 22) As such, the minimum epg value that could be detected by KK was 4 epg and, therefore, individuals only detected by the other parasitological methods were classified as ultra low or <4 epg. Infected individuals detected by the KK method were classified as low (4–99 epg) or medium to high (≥100 epg). The Negative group contained individuals negative for schistosome eggs in any of the parasitological exams, without any other intestinal coinfections, and with eosinophil counts in peripheral blood below 500 per mm^3^. The last group consisted of egg-positive and praziquantel-treated individuals, who were confirmed cured at 30 days post-treatment with 24 KK slides, and remained without reinfection 3 months after chemotherapy with praziquantel, confirmed with Helmintex®, saline gradient, and six KK slides from three fecal samples.

### Fecal Examinations

#### Kato-Katz Method

Before treatment, Helm Test (Biomanguinhos, Rio de Janeiro, Brazil) was performed with three fecal samples collected on consecutive days, as described by Katz et al. (7). From the first sample, up to 14 thick smears were mounted and examined under the microscope. Samples two and three were examined by two slides each. Three months after treatment, three fecal samples from the cured individuals were reexamined, again with two KK slides in each sample, in order to check for possible *S. mansoni* reinfection.

#### Modified Helmintex®

Thirty grams of feces were collected, homogenized in 10% formaldehyde, and purified by successive sieving processes. Subsequently, the sediment was placed in a 15 ml plastic tube and mixed with 5 ml ethyl acetate, according to Ritchie (27). After centrifugation, the sediment was removed from the plastic tube and transferred in a 1.5 ml eppendorf tube, adding 19 μL of paramagnetic beads (Mylteni, Germany). The samples were shook for 1 h on an orbital shaker and then exposed to a strong magnetic field for 3 min (BioMag®, Polysciences, USA). The final sediment trapped, to the walls of the reaction tube, was mixed with a few drops of saline (NaCl 0.9%), transferred onto a glass slide, and examined under the microscope for the presence of *S. mansoni* eggs, as described else where (28, 29).

#### Saline Gradient

Briefly, the apparatus consists of two interconnected cylindrical columns, with the lower column containing 500 mg of feces suspended in 0.9% saline solution. The purification of the fecal suspension with heavy schistosome eggs is guaranteed by a slow flow of a 3% saline solution from the upper column. Approximately 60 min after applying the slow flow of the concentrated saline solution, the suspension in the lower column was discarded and the remaining sediment transferred on microscope slides in order to search for *S. mansoni* eggs (30).

### Measurement of Cytokine and Chemokine Concentrations in Peripheral Blood

In order to search for immunological markers of infection, peripheral blood serum samples from egg-negative endemic individuals, and egg-positive individuals were screened for the following cytokines and chemokines: IL-1β (3.9–500 pg/ml), IL-6 (4.68–600 pg/ml), TNF-α (7.8–1,000 pg/ml), IL-27 (78.13–10,000 pg/ml), CXCL10 (15.63–2,000), CCL3 (3.9–500 pg/ml), IL-17A (7.8–1,000 pg/ml), IL-10 (15.63–2,000 pg/ml), IL-5 (11.72–1500 pg/ml), IL- 13 (46–6,000 pg/ml), IL-33 (11.72–1,500 pg/ml), pg/ml), CCL5 (7.8–1,000 pg/ml), CCL11 (7.8–1,000 pg/ml), and CCL17 (3.9–500 pg/ml). The assays were run in duplicate, final concentrations extrapolated from a standard curve, and were expressed in pg/ml, according to the manufacturer's instructions (Duo Set kits, R&D Systems, USA).

Briefly, Costar half area microplates (Corning, USA) were sensitized with the respective capture antibodies, specific for each cytokine and chemokine and, subsequently incubated overnight at room temperature (RT). On the following day, after a washing step with PBS/ Tween-20 (0.05%), the microplates were blocked with diluent solution (PBS/ 1% BSA) and incubated for 1 h at RT. After another washing step, diluted serum samples (1:2 in diluent solution) and standards were added and incubated for 2 h at RT. Subsequently, plates were washed (5x), incubated with the diluted and biotinylated detection antibodies, and incubated for another 2 h at RT. Thereafter, plates were washed again (5x) and incubated for 20 min with streptavidin-horseradish peroxidase (1:200 in reagent diluent) at RT in the dark. After a last washing step (5x), substrate solution (1:1 H_2_O_2_ and tetramethylbenzidine solution) was added and plates incubated for 20 min. Finally, stop solution (4N H_2_SO_4_) was added and the color reaction read in an automated ELISA reader at 450 nm (Molecular Devices, Versa Max microplate reader). Sample concentrations were extrapolated from the standard curve, using a 4-point curve-fitting program (Softmax Pro 6.4), and considering the sample dilutions.

### Frequency Profiles of Responders for Cytokines and Chemokines

Additional analyses of cytokine and chemokine data included frequency data of responders in each group of individuals, in order to check whether the response pattern was different between the infection groups and in comparison with egg-negative individuals and whether it changed after treatment in each infection group.

For this purpose, each individual cytokine and chemokine concentration was evaluated and defined as a positive response (responder), if the concentration showed a considerable value above the lowest detectable value of the standard curve of each of the different cytokines and chemokines. For IL-β, TNF-α, IL,5, IL-6, IL-13, CCL3, CCL11, CCL17 was 10 pg/ml. For IL-10, IL-17A, IL-27 the cut-off was set to 50 pg/ml and for CXCL10, CCL5, and IL-33 it was set to 100 pg/ml (Table S4).

### Data Analysis

Statistical analysis was performed using the GraphPad Prism 6.0 software package (GraphPad Prism, USA) and Excel. Initially, a descriptive analysis was performed (median and interquartile ranges). Subsequently, the variables were analyzed with specific tests according to the data distribution and compared between the different groups. The normal distribution of the continuous data was verified and discarded through the Shapiro-Wilk test. Non-parametric data were compared between groups, using Kruskal-Wallis test and Dunn's post test. The analyses of paired samples before and after treatment were done with the Wilcoxon test. Categorical variables were compared using Pearson's chi square test. Also, associations between different variables were analyzed with Spearman's rank test. The threshold for statistical significance was set to *p* ≤ 0.05.

## Results

### Parasitology

As described above, we analyzed blood samples from 139 individuals, of which 113 were detected as egg-positive for *S. mansoni* in stool samples by any of the three parasitological methods. Twenty-six individuals were found egg-negative for all of the before-mentioned methods, were negative for all other intestinal protozoan or helminth parasite, and did not show any significant peripheral blood count alterations, which might implicate any other state of infection or clinical condition.

Descriptives of median age, gender distribution, and presence of co-infections in each group were included in (Table 1). There was no significant bias in gender distribution and age between the different infection groups. Although, the egg-negative group consisted of an elevated percentage of male individuals and the group with highest parasite load (≥100 epg) presented the lowest median age. Of the 113 individuals diagnosed as egg-positive for *S. mansoni*, 102 individuals were classified as with a low or ultra low parasite load (Table 1), which represented 90.1% of the egg-positive population. The group with elevated parasite load (≥100 epg) consisted of 11 individuals, of which 7 individuals (6.2%) were considered with a median (100–399 epg) and only four individuals (3.5%) considered with a high parasite load (≥400 epg).

**Table 1:** Characterization of the study groups according to copro-parasitological results in relation to *S. mansoni* infection: Group <4 epg (*n* = 42), Group 4 to 99 epg (*n* = 60), Group ≥100 epg (*n* = 11), and Negatives (*n* = 26). Indicated for each group are sex distribution, median age, parasite load, and presence of co-infections.

**Infection group**	**<4 epg** ***n* = 42**	**4–99 epg** ***n* = 60**	**≥100 epg** ***n* = 11**	**Negative #** ***n* = 26**
Sex male and female (n)	19/23	27/33	6/5	16/10
%	45.2/54.7	45/55	54.5/45.4	61.5/38.4
Age median (IQR)^*^	41.50 (16.5/54.2)	34.50 (19/49.7)	17 (12/33)	40.5 (25/54.7)
Median epg^**^ (IQR)	§	4.5 (4/11)	248 (160/956)	°
Mono-infections with *S. mansoni* (%)	20/42 (47.6%)	30/60 (50%)	5/11 (45.5%)	°
Coinfections with *S. mansoni* and other helminths (%)	7 (16.7%)	1 (1.7%)	2 (18.2%)	°
Coinfections with *S. mansoni* and other protozoa (%)	15 (35.71%)	27 (45%)	2 (18.2%)	°
Coinfections with *S. mansoni* and other helminths and protozoa (%)	°	2 (3.3%)	2 (18.2%)	°

* (IQR) interquartiles range;

** *epg, eggs per gram of feces; #, Group negative, negative results in all parasitological examinations; without co-infection with protozoa (intestinal), § The load was not quantified °without co-infection with protozoa and geohelminths*.

Roughly 50% of schistosome-infected individuals harbored a *S. mansoni* mono-infection in the different infection groups. On the other hand, co-infections with *S. mansoni* and especially with protozoan parasites were common in all infected individuals, but other intestinal helminth infections were not frequently found in this area (Table 1).

**Figure 1:**
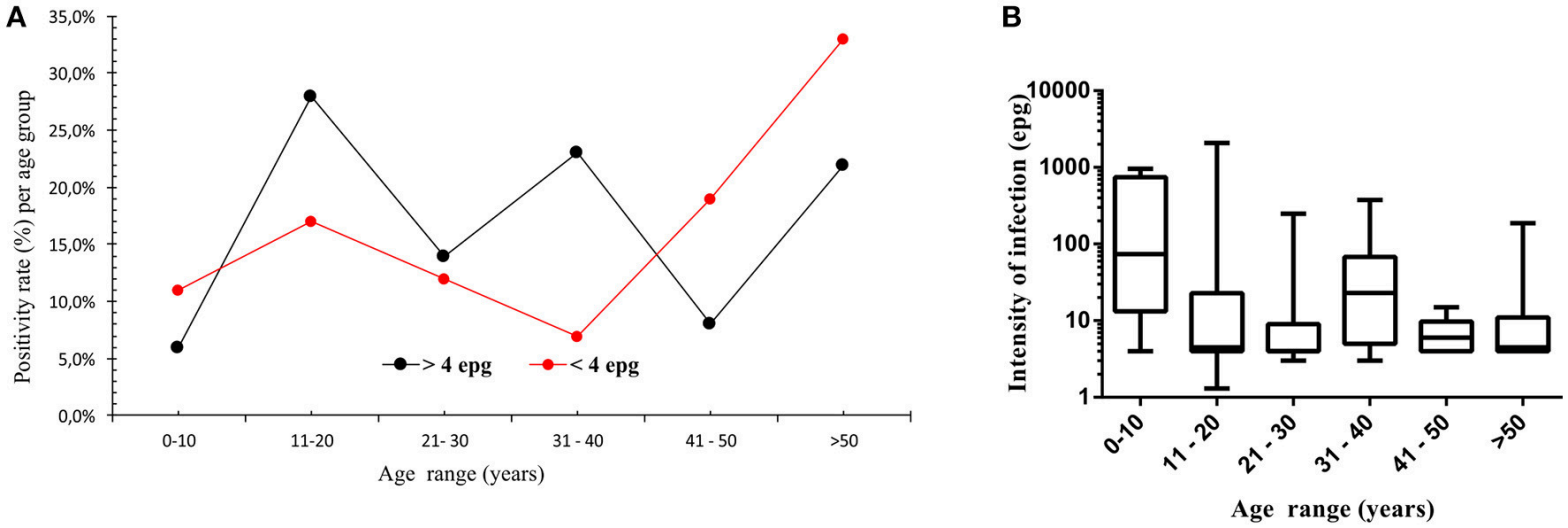
Positivity rate for *S. mansoni* infection per age group **(A)**, indicated for individuals detected by quantitative Kato-Katz method (black squares, >4 epg, *n* = 71) and for individuals detected by other qualitative parasitological methods, such as Helmintex® and saline gradient (red squares, <4 epg, *n* = 42). In **(B)**, median intensity of infection and interquartile ranges per age group, indicated as eggs per gram of feces and determined by the Kato-Katz method for the different age groups (*n* = 71).

In Figure 1A the *S. mansoni* positivity rate is plotted for the different age groups and is divided into individuals who were readily detected by KK exams and individuals who were diagnosed by alternative parasitological methods, e.g., individuals considered as with an ultra low parasite load. KK results indicated the highest positivity rate in individuals during their second decade of life, whereas individuals diagnosed by the alternative methods had elevated and highest positivity rates during their first two decades and in the older age groups, respectively (Figure 1A). Evaluating the intensity of infection determined by KK, no significant differences resulted between the different age groups, but a highest median value resulted in the youngest age group (Figure 1B).

**Figure 2:**
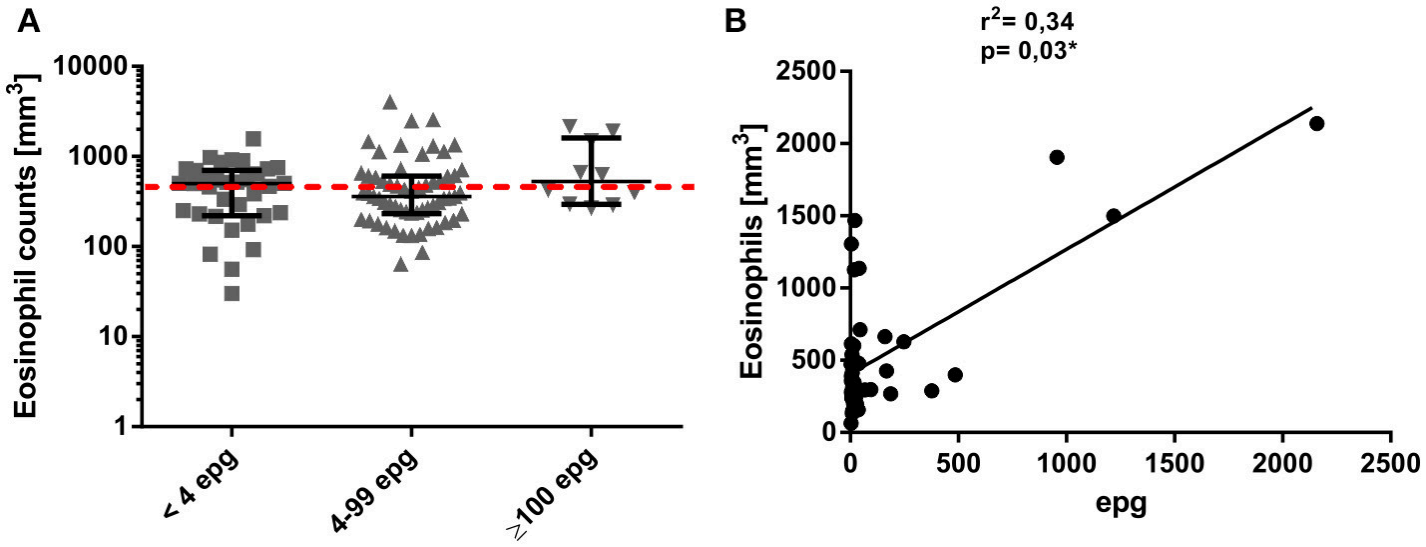
Individual eosinophil counts (mm^3^) in different *S. mansoni* infection groups **(A)**. Groups were classified according to their individual egg counts by six Kato-Katz slides into median to high (≥100 epg) or low (4 to 99 epg) parasite load, or into <4 epg, according to more sensitive, qualitative detection methods. The dotted red line indicates the limit of normal eosinophil counts in clinical samples (range 0–500 mm^3^). Association between peripheral blood eosinophil counts and egg counts per gram of feces, as determined by Kato-Katz **(B)**.

When peripheral blood eosinophil counts were compared between the different infection groups, no significant differences resulted between individuals with an ultra low, low, or median to high parasite load (Figure 2A). Interestingly, in these infection groups the percentage of individuals who presented with peripheral blood eosinophilia was only 42.9, 33.3, and 50.0% in individuals classified as with ultra low, low, or median to high parasite load, respectively. Nevertheless, for egg-positive individuals with a quantitative KK result, a weak but significant correlation (*p* = 0.03) between eosinophil counts and epg values was observed (Figure 2B).

### Cytokine and Chemokine Profile in Sera From Infected and Non-Infected Individuals Before Treatment

**Figure 3:**
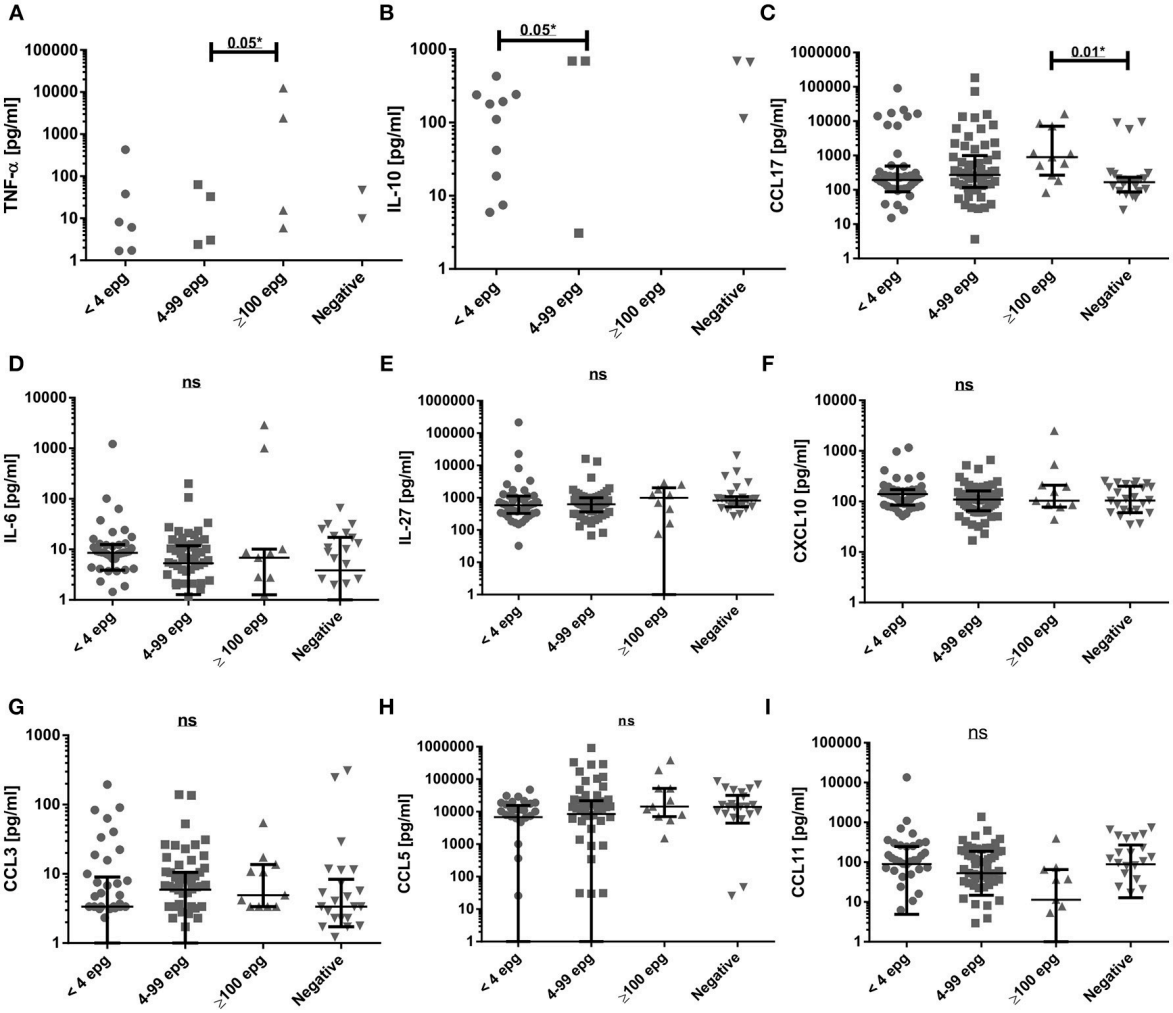
Comparison of serum cytokine and chemokine concentrations (pg/ml) in different *S. mansoni* infection groups. Groups were classified according to their individual egg counts into median to high (≥100 epg; *n* = 11) or low (4 to 99 epg; *n* = 60) parasite load, or into <4 epg (*n* = 42) and compared with egg-negative individuals (*n* = 26). Shown are results for TNF-α **(A)**, IL-10 **(B)**, CCL17 **(C)**, IL-6 **(D)**, IL-27 **(E)**, CXCL10 **(F)**,CCL3 **(G)**, CCL5 **(H)**, CCL11 **(I)**. Indicated were median values and 25 and 75% interquartile ranges and data were analyzed with Kruskal-Wallis test and Dunn's post-test. Not significant differences (ns: *p* ≥ 0.05) and significant differences between groups (^*^p ≤ 0.05) were indicated.

In order to search for serological markers of infection, we evaluated the immunological profile of type 1, type 2, inflammatory markers, and of regulatory chemokines and cytokines in individuals with different parasite loads and compared them with non-infected individuals. Initially, the raw cytokine and chemokine concentrations were compared between the different groups. Serum concentrations of some inflammatory (IL-1β and IL-17A) and type 2 cytokines (IL-5, IL-13, IL-33) were very low and were not shown (Table S1). With a few responders in each infection group, the serum concentrations of the cytokines TNF-α and of IL-10 showed significant differences between the groups ≥100 epg and 4–99 epg for TNF-α and between 4–99 epg and <4 epg for IL-10 (*p* ≤ 0.05) for each comparison, (Figures 3A,B). The most striking differences were observed for type 2 CCL17. Here, we measured a significant higher serum concentration (*p* ≤ 0.05) in the group ≥100 epg, when compared with the non-infected controls (Figure 3C). The cytokines and chemokines IL-6, IL-27, CXCL10, CCL3, CCL5, and CCL11 were readily detected in almost all serum samples. However, no significant differences were observed between the different infection groups (Figures 3D–I).We further checked whether co-infections with protozoan parasites and/or intestinal helminths, as described in (Table 1), might have influenced cytokine and chemokine concentrations in sera. Only a few alterations between mono- and co-infected individuals in each of the schistosome infection groups were measured. In the group <4 epg, co-infected individuals showed significantly (*p* ≤ 0.01) higher IL-10 concentrations when compared with mono-infected individuals, whereas, IL-13 was significantly elevated (*p* ≤ 0.01) in mono- as compared with co-infected individuals. For the group 4–99 epg, significantly higher (*p* ≤ 0.01) concentrations of inflammatory IL-6 were observed in co-infected when compared with mono-infected individuals (Table S2).

When the cytokine and chemokine concentrations in sera were correlated with the individual parasite load of all egg-positive individuals diagnosed by the KK method, significant positive correlations between epg values and IL-1β (*p* = 0.006) and with TNF-α (*p* = 0.002) were detected (Table 2, Figure S1).

**Table 2:** Correlations between cytokine and chemokine concentrations in peripheral blood and *S. mansoni* egg counts (epg), as determined in individuals by the Kato-Katz method (>4 epg).

>4 epg^*^		
**Cytokines and chemokines**	**Correlation coefficient**	
	***r***^**2**^	***p***^**a**^**-value**
IL-1β	0.32	0.006^**^
IL-6	0.002	−0.98
TNF-α	0.36	0.002^**^
IL-27	0.2	0.07
CXCL10	−0.004	0.97
IL17A	0.13	0.26
IL-10	−0.13	0.26
IL-5	−0.04	0.7
IL-13	0.08	0.52
IL-33	0.15	0.25
CCL11	−0.12	0.33
CCL17	0.11	0.35
CCL5	−0.028	0.83
CCL5	0.07	0.59

* epg, eggs per gram of feces;

a Spearman's rank test with significance of ^*^p ≤ 0.05. Bold values indicate significant correlations between cytokine/chemokine concentrations and egg counts (

** *p < 0.01)*.

### Cytokine and Chemokine Profile in Sera From Formerly Infected Individuals Three Months Post-Treatment

**Figure 4:**
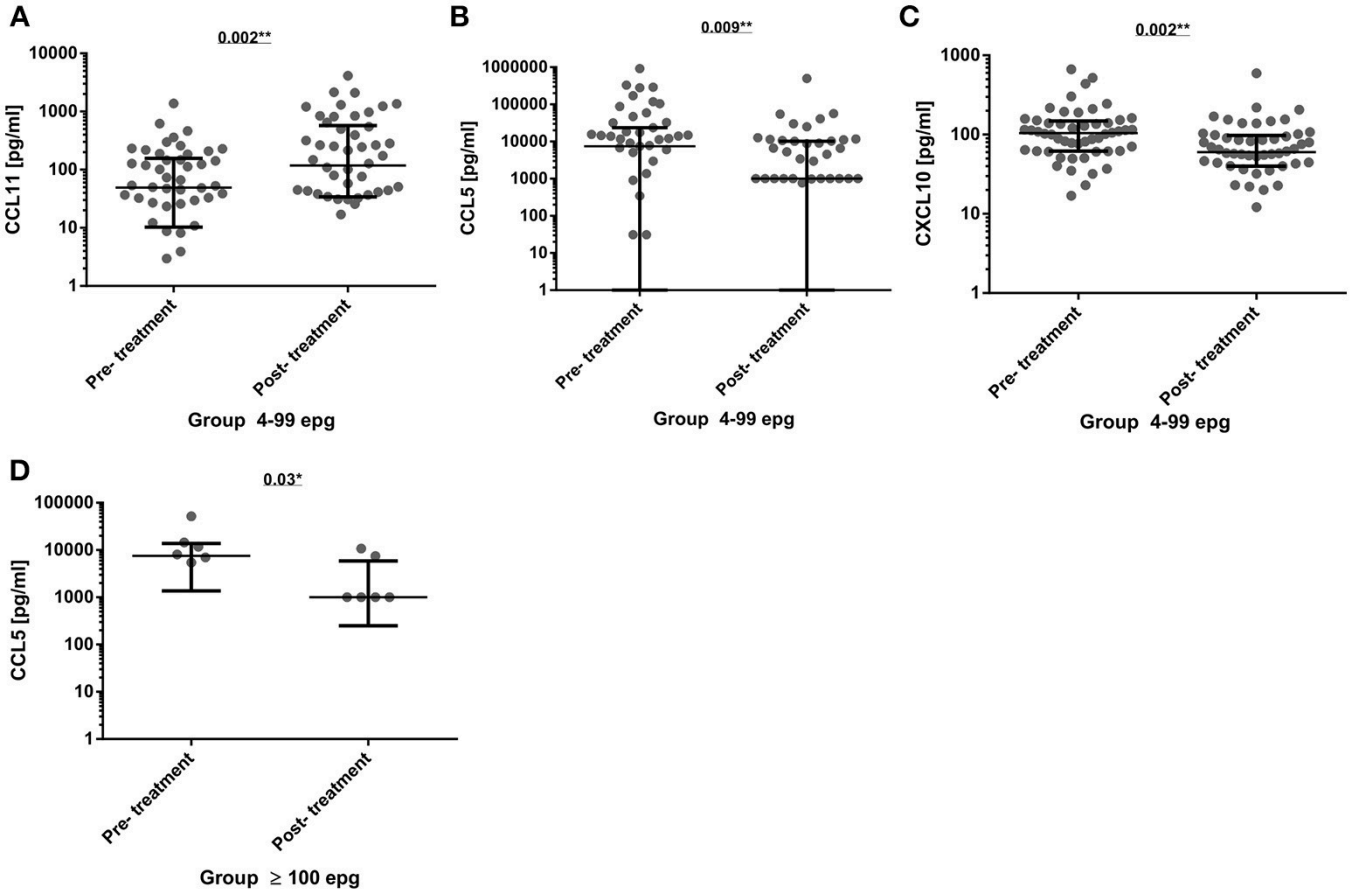
Comparison of serum cytokine and chemokine concentrations (pg/ml) before (pre-treatment) and 3 months after chemotherapy (post-treatment) in group 4–99 epg (*n* = 51) for the chemokines CCL11 **(A)**, CCL5 **(B)**, and CXCL10 **(C)**. And in group ≥100 epg (*n* = 8) for the chemokine CCL5 **(D)**. The data were analyzed by the paired Wilcoxon test and significant differences and *p*-values were indicated in each graph. ^*^p < 0.05; ^**^p < 0.01.

The infected individuals were treated with praziquantel, treatment efficacy was confirmed at 1-month post-treatment, and those individuals were offered another extensive parasitological examination at 3 months post-treatment in order to search for reinfection. The immunological profile of 78 individuals, who were confirmed as egg-negative (without reinfection) was re-evaluated. Most of the cytokines and chemokines did not show any significant alterations at 3 months post-treatment when compared with pre-treatment serum concentrations (Table S3). However, in the 4–99 epg infection group, there was a significant increase in CCL11 (*p* = 0.002) 3 months post-treatment, whereas, CCL5 and CXCL10 were significantly decreased (*p* = 0.009 and *p* = 0.002, respectively). Also, a significant reduction of CCL5 levels (*p* = 0.03) was measured in treated individuals with a former egg load of 100 or more (Figures 4A–D).

### Frequency of Cytokine and Chemokine Responders in Each Infection Group Before and 3 Months After Treatment

**Figure 5:**
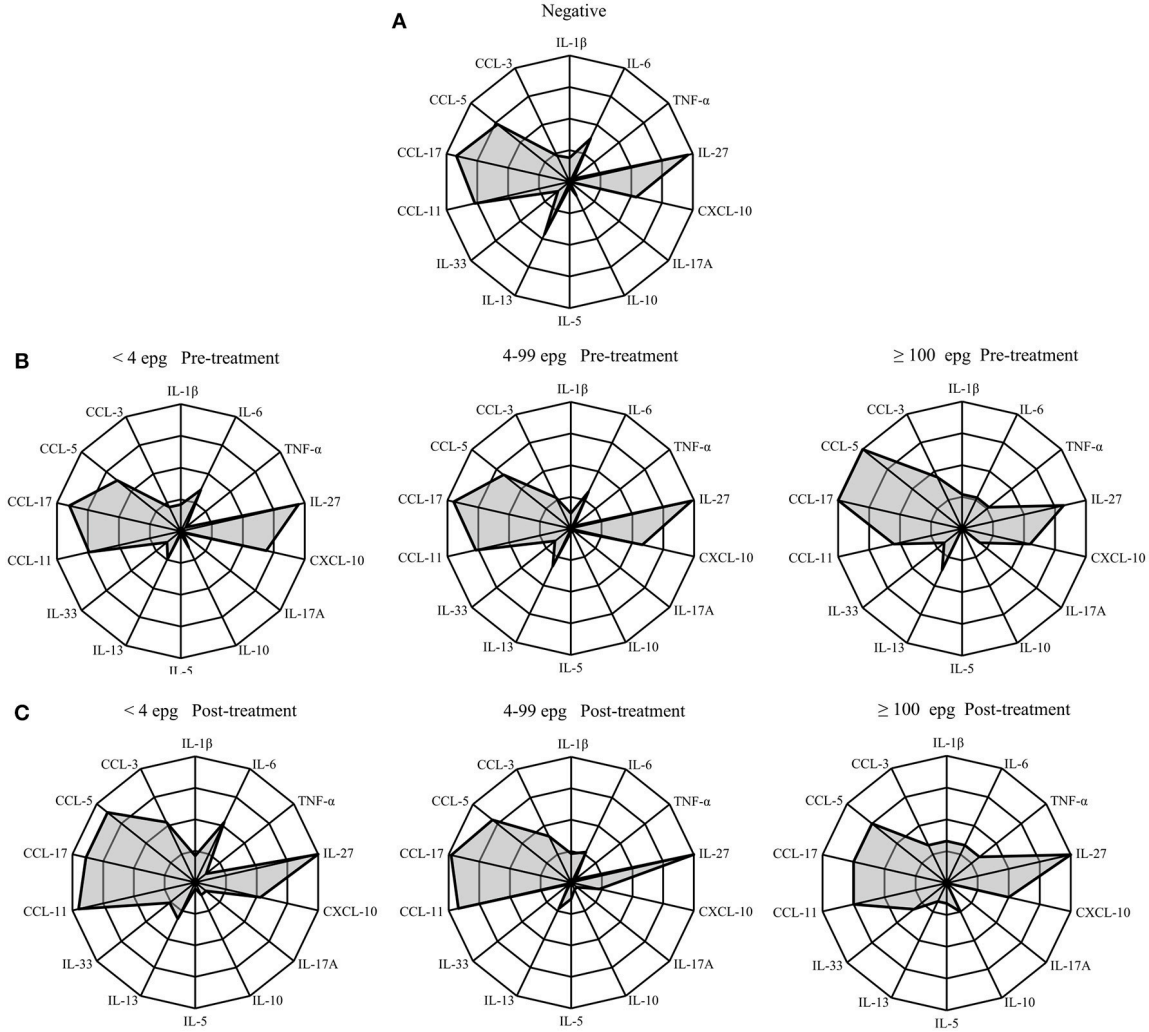
Radar profile for cytokines and chemokines in individuals infected with *S. mansoni*, compared with egg-negative individuals at pre-treatment **(A,B)**, and 3 months post-treatment **(C)**. Infection groups were classified according to different parasite loads in <4 epg, 4–99 epg and ≥100 epg. Indicated radar zones correspond to 25, 50, 75, and 100 percent values. Radar indicates percentage of responders in each infection group before and 3 months post-treatment and in comparison with endemic egg-negative individuals.

In order to visualize the immunological profile in each infection group before and after treatment, we put together radar diagrams with the percentages of responders in each infection group for the different cytokines and chemokines under investigation (Figure 5 and Table S4).

At pre-treatment, the chemokines CCL5, CCL11, and CCL17 were readily detected in all infection groups and the frequencies of responders were similar to the egg-negative individuals (Figures 5A,B). Also, IL-27 was measured in serum samples from more than 95% of all individuals of the different infection groups with the exception of individuals with an egg load of 100 epg or more. In this infection group the frequency of IL-27 was reduced to 82%. Likewise, the frequency of CCL11 (eotaxin-1) was reduced from around 75% in the other infection groups to 55% in individuals with an elevated parasite load (≥100 epg). On the other hand, the frequencies of responders for CCL5 and CCL17 increased to 100% in this group and, together with IL-6, TNF-α was detectable in more than 25% of infected individuals (Figure 5B). The statistical analysis of frequencies of responders in each infection group and for each immunological marker are shown in Table S4 and frequencies of responders for TNF-α and IL-17A were significantly elevated (*p* = 0.016 and *p* = 0.048, respectively) in the infection group with highest parasite load (≥100 epg). Also, CCL5 showed a tendency for increased frequencies in this group, however, differences did not reach the significance level (Table S4).

Three months after treatment with praziquantel, the groups with lower parasite load (<4 epg and 4–99 epg) showed similar alterations in cytokine and chemokine frequencies, whereas, the highest infection group (≥100 epg) showed a different pattern (Figures 5B,C). Especially the chemokines CCL3, CCL5, and CCL11 increased in the lower infection groups 3 months after treatment. Opposite to that, a prominent increase in frequencies for CCL3, CCL5, and CCL17 was measured in individuals with ≥100 epg, whereas, eotaxin-1 (CCL11) was reduced after treatment. Also, in individuals with highest parasite loads (≥100 epg) frequencies for IL-27 and for IL-10 increased after treatment to 100 and 25%, respectively. Finally, throughout all infection groups, the frequencies of CXCL10 were reduced after treatment (Figure 5C).

## Discussion

The main goal of the present study was to search for infection markers in serum from individuals with asymptomatic *S mansoni* infection and ultra low parasite load (<4 epg). Individuals with such a low parasite burden are hardly detected by the control programs, using the commonly applied KK method. However, by combining several parasitological methods, we were able to show that a considerably higher percentage of the population was actually infected with schistosomes (13). The study area matches the present epidemiological situation in many endemic Brazilian regions, where frequent treatment cycles have led to a considerable reduction of clinical cases and morbidity and reductions in the individual and community parasite loads (5).

Nowadays, most of the BSCP controlled endemic areas for intestinal schistosomiasis have populations with median to low individual parasite loads (31), thus, this turns precise individual diagnosis much more difficult. As a consequence, more sensitive direct or indirect methods or combination of methods are urgently needed for diagnosis (32). One alternative are indirect serological methods, which are capable to detect acute and chronic infections, show a high sensitivity, but usually have problems with the specificity, due to other cross-reactive infections (10). Therefore, several groups have been searching for other indirect immunological markers, which could indicate a present schistosome infection in the absence of extensive parasitological exams (26, 33, 34).

In our study, the first step was to elucidate the infection profile on a populational level and in the different age groups. If the diagnostic method of choice was KK, the infection profile showed the highest infection rate in children and young adults from 11 to 20 years of age, which was similar to earlier studies (30, 31). Interestingly, when more sensitive parasitological methods were used, the infection profile was somewhat inverted. Now, the youngest and the oldest age groups showed elevated and the highest rates of infection, thus, showing their relevance and contribution for maintaining the parasitic cycle and continuous transmission. In the youngest age group, a considerable population with adult parasite worms and increased parasite loads are supposed to build up slowly with increasing exposure during the first years of their life and, therefore, eggs might not be readily detected in common stool exams (35–37). On the other end of the age scale, reduced parasite loads in elderly individuals might be explained by more effective immune responses and reduced reinfection rates, reduced exposure due to altered habits and/or by aging worms and reduced fertility of female parasites (15, 38, 39).

Alterations in peripheral blood leukocyte counts and especially elevated eosinophil counts are considered a hallmark of helminth infections (40). However, in our study we were able to detect peripheral blood eosinophilia only in 33–50% of our infected individuals, depending on the infection group, which would not classify eosinophil counts as a good and mandatory infection marker. For example, for experimental *S. mansoni* infections in mice it was shown that activation, recruitment and granuloma composition of eosinophils vary to a considerable degree, depending on the phase of infection and time post-infection (41) Nevertheless, in individuals with quantitative KK egg counts, peripheral blood eosinophilia correlated to a significant degree, as was already observed in former studies (42, 43).

Since the major goal was the search of detectable infection markers in the peripheral blood of individuals with very low parasite loads, we tried to identify cytokines and chemokines, which could readily be detected in serum samples. Generally, during the migration and acute phase of infection it was shown that schistosome antigens induce a host immune response in humans, which is characterized by increased secretion of type 1 cytokines, such as IL-2 and IFN-γ (14, 15, 20, 44–46). Later on, during the chronic phase of infection, this type 1 profile is gradually modulated to a type 2 profile (15, 20, 45, 47), with the major contribution of IL-10 (20, 39, 45, 48) and especially T regulatory cells as its source (16, 49). In addition, in patients with confirmed clinical alterations the immunological profile was shown to be driven by the secretion mainly of type 2 IL-13 (18, 50, 51), and TNF-α (52) and an upregulation of Th 17 cells (53). All of the before mentioned studies on the immune response in human schistosomiasis were based on results obtained by *in vitro* stimulation of peripheral blood mononuclear cells (PBMC) or whole blood cultures with egg or adult worm antigens from schistosomes.

Here, we searched for circulating infection markers in sera from individuals with different infection intensities, before and 3 months after treatment with praziquantel, and compared cytokine and chemokine concentrations and their frequencies with parasitologically well-confirmed and egg-negative individuals from the same endemic area.

A panel of pro-inflammatory, type 1, type 2, Th 17, and regulatory cytokines and chemokines were measured in serum samples. First, we tried to correlate any of the immunological markers with egg counts, but only serum IL-1β and TNF-α concentrations weakly correlated with KK epg values throughout the infection groups, which corroborated the results obtained by Coutinho et al. from individuals with elevated parasite loads and/or clinical manifestations (23).

As already shown before (26), most of the pro-inflammatory markers, such as IL-1β and IL-6, were only detected at low concentrations in the different infection groups. However, TNF-α serum levels in individuals with a medium to high parasite load (≥100 epg) were significantly higher than in the group with a low intensity of infection (4–99 epg). In this case, this might be an indicator of pathological changes in apparently asymptomatic individuals, though they were only observed in a restricted number of individuals. However, thorough clinical and ultrasound examinations were not within the scope of the present study and were not done. In a former study (21), such elevated TNF-α levels were not found in schistosome-infected individuals even with a median to high parasite load. This might be due to the presence of co-infections with intestinal nematodes and might be explained by a deviation to a type 2 immune response, as measured by increased CCL11 and CCL17 concentrations in co-infected individuals (21). In addition, IL-17A was hardly detected in any of the infection groups, which would be more indicative of chronic inflammatory processes (54) or, in schistosomiasis, be more indicative of severe liver pathology (53, 55, 56). As already shown for multiple helminth infections and at increased parasite loads (26), serum CCL17 concentrations were highest in the infection group with ≥100 epg, but in the infection groups with low or ultra-low parasite loads, the CCL17 concentrations did not differ from non-infected individuals. Also, CCL11 (eotaxin-1) was not found significantly elevated in the infection groups, when compared with non-infected individuals. This is in contrast to what was previously found in individuals co-infected with *S. mansoni* and with geohelminths (26), and, again, might be explained by low parasite loads and absence of co-infections with intestinal helminths in the present study. Therefore, the results of serum CCL11 and CCL17 concentrations did not support previous findings that these chemokines could also be used as infection markers in individuals with diminished parasite loads. On the other hand, type 1 markers and pro-inflammatory markers, such as CXCL10 (57) and IL-27 (58), were readily detected in serum samples, but throughout all groups and without significant differences between infected and egg-negative individuals. This fact, also might argue against acute infections or severe schistosome-induced pathologies in the examined individuals, as found in other studies (18, 52, 59, 60). They are rather indicative of balanced type 1/type 2 immune responses during asymptomatic chronic infections (15). Interestingly, a most recently published study on chemokines in plasma samples of individuals with fibrotic lesions but with low parasite loads, also did not reveal any significant differences in concentrations of several chemokines between groups of infected individuals and egg-negative controls. However, individual chemokine concentrations, such as CCL3, CCL7, CCL24 (eotaxin-2), Macrophage Migration Inhibitory Factor (MIF), and soluble TNF-α receptor 1, were positively associated with *Schistosoma*-related liver fibrosis (61). As far as the comparison of frequencies of the different cytokines and chemokines between infection groups and egg-negative individuals are concerned, we found a very similar pattern in the groups with ultra-low and low parasite loads, when compared with non-infected individuals. Only the group with moderate to high parasite loads (≥100 epg) showed alterations, which would be indicative of inflammatory processes. However, other biomarkers who were found to be associated with granuloma size and severity of the disease in animal models and in humans, were only found at low frequencies. From the cytokines and chemokines found in serum samples with high frequencies, IL-27 is considered a pro-inflammatory cytokine, which is able to regulate Th1, Th2, and Th17 responses, as shown in experimental infections (62) The chemokines CCL5, CCL11, and CCL17 were also found in high frequencies, especially in the group with the highest parasite loads (≥100 epg). In other studies it was shown that these chemokines were important to attract eosinophils and to inhibit severe disease. On the other hand, especially CCL3 and CCL5 were shown to be elevated in schistosomiasis patients with severe disease (63–66). Three months after treatment, the frequencies in the cytokine and chemokine profiles again were somewhat different for the infection groups with low parasite loads (<4 epg and 4–99 epg) when compared with individuals with medium to high parasite loads. This difference might be explained by the different amount of released antigens after chemotherapy, or by more profound immuno-modulatory and regulatory mechanisms in individuals with a higher parasite load. However, this was not addressed in the present study and could be part of future research.

In conclusion, we believe in the importance to search for alternative markers of infection in human schistosomiasis, in order to overcome shortcomings of correct parasitological diagnosis in individuals with reduced parasite loads. However, the immunological alterations in peripheral blood (serum or plasma) of such individuals are subtle and might not be measurable by the common immunological assays, instead, they only become more obvious in individuals with medium to high parasite loads and/or advanced pathology.

## Author Contributions

SG: conception and design of the work, acquisition of data and field work, analysis and interpretation of the work, drafted the work, and critically revised the work. VC: acquisition of data and field work, analysis and interpretation of the work, drafted the work, and critically revised the work. JR: acquisition of data and field work, analysis and interpretation of the work, critically revised the work. DC: acquisition of data and field work, analysis and interpretation of the work, critically revised the work. SR: acquisition of data and field work, analysis and interpretation of the work, critically revised the work. FM: acquisition of data and field work, analysis and interpretation of the work, critically revised the work. DS: acquisition of data and field work, analysis and interpretation of the work, critically revised the work. MR: acquisition of data and field work, analysis and interpretation of the work, critically revised the work. DN-C: conception and design of the work, analysis and interpretation of the work, drafted the work, and critically revised the work.

### Conflict of Interest Statement

The authors declare that the research was conducted in the absence of any commercial or financial relationships that could be construed as a potential conflict of interest.
