# Supplementary material for: Systemic Cytokine and Chemokine Profiles in Individuals With Schistosoma mansoni Infection and Low Parasite Burden
Source: Front Immunol. 2018 Dec 18;9:2975. doi: 10.3389/fimmu.2018.02975 (PMC6305627; doi:10.3389/fimmu.2018.02975)

**Supplementary Material:**

**Systemic cytokine and chemokine profiles in individuals with *Schistosoma mansoni* infection and low parasite burden**

Vanessa N. Castro^1^, Jailza L. Rodrigues^1^, Diogo T. Cardoso^1^ ,Samira D. Resende^1^, Fernanda C. Magalhães^1^, Dayane C. Souza^1^, Maira H. Requeijo^2^, Deborah A.Negrão-Corrêa^1^, Stefan M. Geiger^1^*

*e-mail address:* stefan.geiger76@gmail.com (S. M. Geiger).

**Table S1-** Cytokine and chemokine concentrations (pg/ml) in serum from *Schistosoma mansoni-*infected individuals and egg-negative controls. Infection groups were classified according to their quantitative and qualitative parasitological exams in ≤ 4 epg (ultra-low), 4-99 epg (low), and ≥ 100 epg (medium to high parasite load). Indicated are median values for each parameter in each group plus interquartile ranges.

**Table S2-** Cytokine and chemokine concentrations (pg/ml) in serum from *Schistosoma mansoni*-infected individuals, subdivided into mono- and co-infected individuals with intestinal protozoa and/or geohelminths. Infection groups < 4 epg, monoinfected (n=20) and coinfected (n=22); group 4 - 99 epg, monoinfected (n=30) and coinfected (n=30). Indicated are median values for each parameter in each subgroup plus interquartile ranges.

**Table S3-** Paired cytokine and chemokine concentrations (pg/ml) in serum from *Schistosoma mansoni*-infected individuals pre- and three months post-treatment with praziquantel. Infection groups contained paired samples from < 4 epg (n=19), 4 – 99 epg (n=51) and ≥ 100 epg (n=9). Indicated are median values for each parameter in the groups plus interquartile ranges at pre- and three months post-treatment.

**Table S4-** Frequency (%) of cytokine and chemokine responders in the peripheral blood of different *Schistosoma mansoni* infection groups and compared with egg-negative individuals. Infection groups were classified according to their quantitative and qualitative parasitological exams in ≤ 4 epg (ultra-low), 4-99 epg (low), and ≥ 100 epg (medium to high parasite load)

**Figure S1** Correlation between peripheral blood IL-1β **(A)** and TNF-α **(B)** serum concentrations (pg/ml) and *Schistosoma mansoni* egg counts, as determined by the Kato-Katz method.


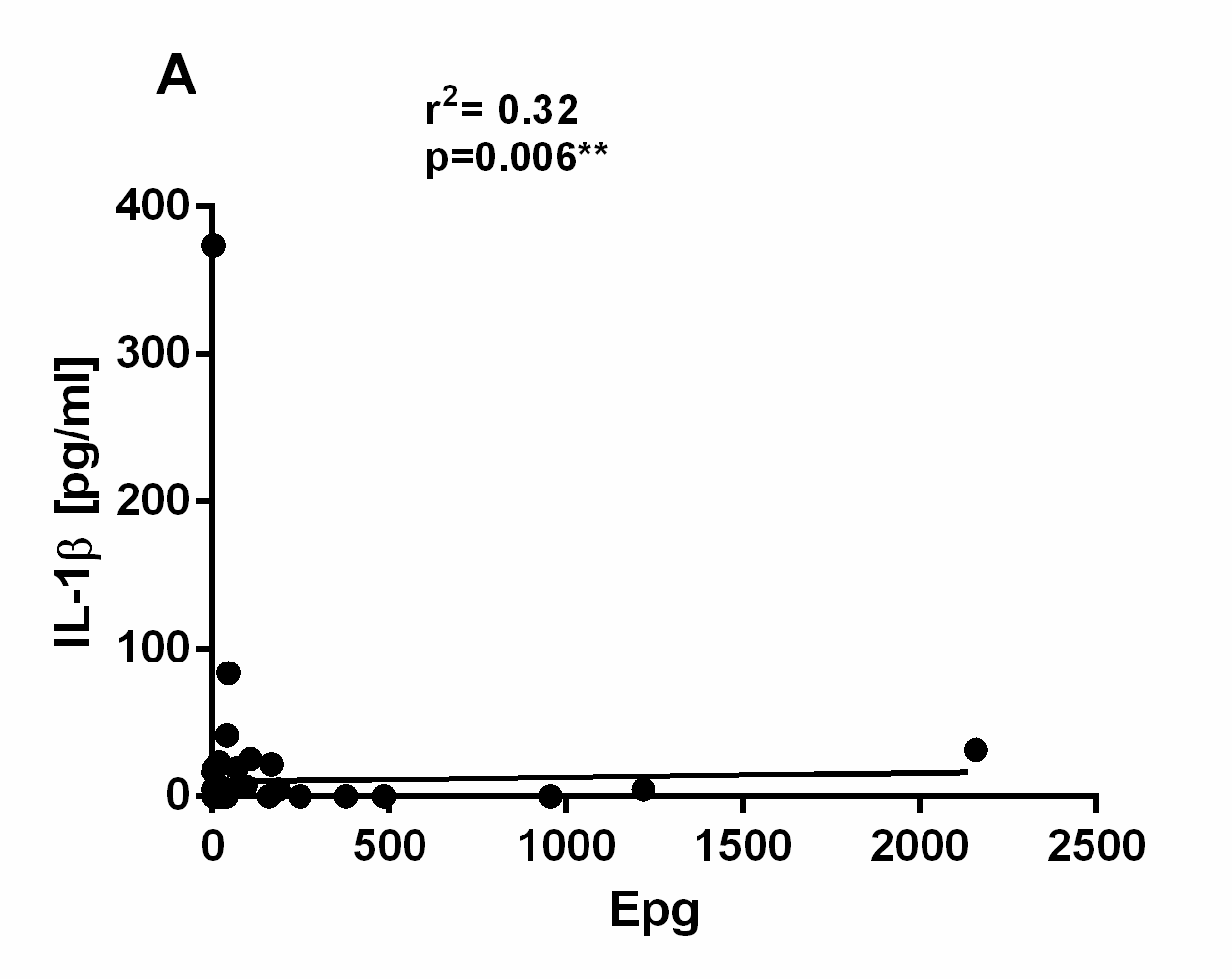

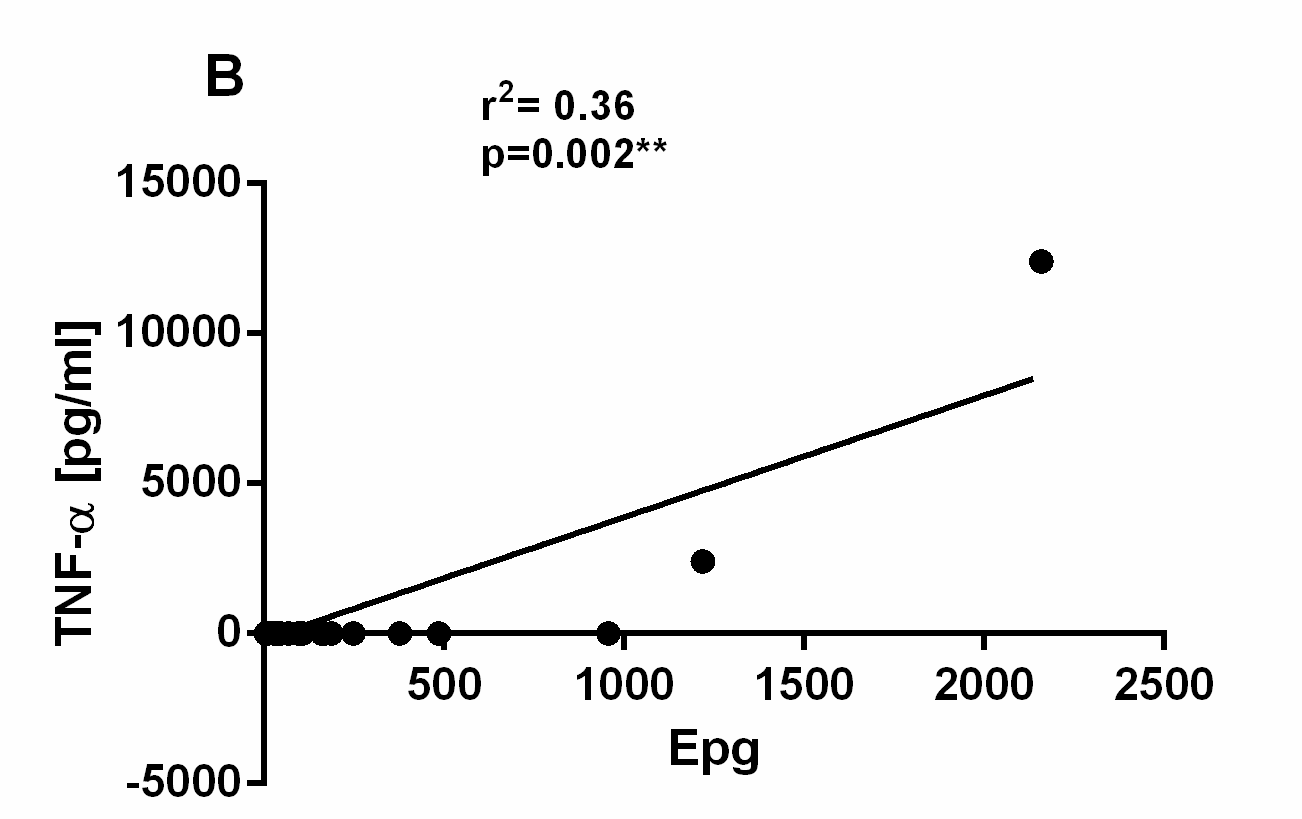

Supplement: Supplementary file 1 [file Data_Sheet_1.docx]
